# Evolution in Documented Goals of Care at End of Life for Adolescents and Younger Adults With Cancer

**DOI:** 10.1001/jamanetworkopen.2024.50489

**Published:** 2024-12-19

**Authors:** Rosemarie Mastropolo, Colin Cernik, Hajime Uno, Lauren Fisher, Lanfang Xu, Cecile A. Laurent, Nancy Cannizzaro, Julie Munneke, Robert M. Cooper, Joshua R. Lakin, Corey M. Schwartz, Mallory Casperson, Andrea Altschuler, Lawrence Kushi, Chun R. Chao, Lori Wiener, Jennifer W. Mack

**Affiliations:** 1 Division of Population Sciences, Dana-Farber Cancer Institute, Boston, Massachusetts; 2 Department of Pediatric Oncology, Dana-Farber Cancer Institute, Boston, Massachusetts; 3 MedHealth Statistical Consulting Inc, Solon, Ohio; 4 Division of Research, Kaiser Permanente Northern California, Oakland; 5 Department of Research and Evaluation, Kaiser Permanente Southern California, Pasadena; 6 Department of Pediatric Oncology, Kaiser Permanente Southern California, Pasadena; 7 Department of Psychosocial Oncology and Palliative Care, Dana-Farber Cancer Institute, Boston, Massachusetts; 8 Division of Medical Oncology, Kaiser Permanente Northern California, Oakland; 9 Cactus Cancer Society, Oakland, California; 10 Psychosocial Support and Research Program, National Cancer Institute, Bethesda, Maryland

## Abstract

**Importance:**

Little is known about the nature of change in goals of care (GOC) over time among adolescents and younger adult (AYA) patients aged 12 to 39 years with cancer near the end of life. Understanding how GOC evolve may guide clinicians in supporting AYA patients in making end-of-life decisions.

**Objective:**

To assess frequency, timing, and evolution of documented GOC among AYA patients with cancer in the last 90 days of life.

**Design, Setting, and Participants:**

This cross-sectional study included a retrospective review of medical records from the Dana-Farber Cancer Institute, Kaiser Permanente Northern California, and Kaiser Permanente Southern California of AYA patients with cancer who were 12 to 39 years of age at death and who died between January 1, 2003, and December 31, 2019. Data were analyzed from July 1, 2023, through April 30, 2024.

**Exposures:**

Stage IV cancer or stage I-III cancer with new metastasis or recurrence.

**Main Outcome and Measures:**

The primary outcome was documented GOC discussions, categorized by timing before death as initial (>60 days), middle (31-60 days), or late (≤30 days). Goals were classified as palliative, nonpalliative, undecided, or not discussed. Subgroup analysis according to race and ethnicity were also performed.

**Results:**

Among 1929 AYA patients with a mean (SD) age at cancer diagnosis of 28 (8) years, 1049 (54.5%) were female; 5 (0.3%) were American Indian or Alaska Native, 227 (11.8%) were Asian, 157 (8.1%) were Black or African American, 14 (0.7%) were Native Hawaiian or Other Pacific Islander, 1184 (61.4%) were White, 11 (0.6%) were of more than 1 race, 38 (2.0%) were categorized as other race, 293 (15.2%) were without documented race, 514 (26.6%) were Hispanic or Latino, 762 (39.5%) were not Hispanic or Latino, and 653 (33.9%) had no documented ethnicity. Few AYA patients had palliative goals documented in the initial period (139 [7.2%]), increasing to 331 (17.2%) in the middle period and 1113 (57.7%) in the late period. In total, 393 patients (20.4%) transitioned from documented nonpalliative goals in the initial or middle periods to palliative goals by the late period. Many patients had no documented GOC discussion until close to death (initial, 1364 [70.7%]; middle, 969 [50.2%]; and late, 322 [16.7%]). Among the 1929 patients, non-White patients were disproportionately represented among those not having documented GOC discussions (Black, 30 of 157 [19.1%]; Asian, 45 of 227 [19.8%]; and other or undocumented race, 80 of 361 [22.2%]) compared with White patients (167 of 1184 [14.1%]) (*P* < .001) as were Hispanic or Latino patients (116 of 514 [22.6%]) compared with non-Hispanic patients (93 of 762 [12.2%]) (*P* < .001) and individuals with no ethnicity documented (113 of 653 [17.3%]) (*P* < .001).

**Conclusions and Relevance:**

In this cross-sectional study of AYA patients who died of cancer, palliative goals were rarely documented before the last month of life, highlighting the need for timely and ongoing GOC discussions.

## Key Points

**Question:**

How do the goals of care evolve for adolescents and younger adult (AYA) patients 12 through 39 years of age with cancer in the last 90 days of life?

**Findings:**

In this cross-sectional study of 1929 AYA patients with cancer, documented palliative goals of care increased as patients approached death, from 7.2% of patients 61 to 90 days before death to 17.2% 31 to 60 days before death and 57.7% in the last 30 days, with 20.4% of patients transitioning from nonpalliative to palliative goals before death.

**Meaning:**

These findings underscore a need for early and ongoing discussions of goals of care to allow the opportunity to learn of palliative goals of AYA patients with cancer before the late end-of-life period.

## Teaser

This cross-sectional study assesses the frequency, timing, and evolution of discussions and documented goals of care in the last 90 days of life among adolescents and young adults with cancer.

## Introduction

Patient goals are an essential guidepost for care planning when cancer is incurable. Patients who have the opportunity to discuss their treatment preferences are more likely to receive care aligned with their values.^1^ As a result, multiple organizations recommend discussions about goals of care (GOC) for patients living with advanced cancer.^2,3,4,5,6,7,8^

However, many patients do not have GOC discussions, and others have discussions late in their illness course,^1,9,10^ challenging delivery of care that is fully aligned with patient values. Additionally, patient goals may evolve as their prognostic awareness evolves, with changes in functional status and quality of life, with evolving acceptance of their condition and future uncertainties, and as medical options change.^11,12^ Some patients may also experience ambivalence about the best path forward. Aligning care to meet patient needs over this evolution is likely to require continued conversation over time.

Limited literature has addressed the nature of change in GOC over time.^11,12^ To address this gap, we examined evolution in GOC documented in medical records for adolescents and younger adults (AYA) patients 12 to 39 years of age at death with cancer in their last 90 days of life. AYA patients may be particularly prone to shifts in GOC over time given the challenges of contemplating death at a young age. By nature of their developmental phase, AYA patients may also experience more ambivalence about decisions and have a greater tendency to shift goals than older adults with more life experience.^13,14,15,16^ Understanding this evolution may help clinicians best support AYA patients who are weighing care decisions at the end of life.

We examined documented GOC in the last 90 days of life among nearly 2000 AYA patients at 3 sites. Using medical records, we assessed whether GOC discussions were documented, which goals were recorded, and how documented goals evolved over time. We also evaluated factors associated with earlier documentation of palliative goals. Finally, to understand whether evolution in goals was associated with care received, we assessed outcomes of the nature and timing of GOC.

## Methods

The overarching aim of this cross-sectional study was to examine end-of-life care quality among AYA patients with advanced cancer. Some findings have been previously reported,^17,18^ including the prevalence of documented GOC discussions and end-of-life care planning.^9^ The present report builds on those findings to examine the evolution in documented GOC, including trajectories of change in the 90 days before death. The study was approved by the institutional review board for each site with a waiver for the requirement of obtaining informed consent given the retrospective nature of the study and that the participants of interest were deceased. This study follows Strengthening the Reporting of Observational Studies in Epidemiology (STROBE) reporting guidelines.^19^

### Study Setting and Population

We examined medical records of AYA patients with cancer who died after receiving care at the Dana-Farber Cancer Institute (DFCI), Kaiser Permanente Northern California (KPNC), or Kaiser Permanente Southern California (KPSC). The DFCI is an academic cancer center; KPNC and KPSC are integrated health plans and care delivery systems. The DFCI and KPNC included patients who died between January 1, 2003, and December 31, 2019; the KPSC included patients who died between January 1, 2009 and December 31, 2019, to match years of available electronic records. Our data analysis was completed between July 1, 2023, and April 30, 2024.

Each site used electronic health record (DFCI) or cancer registry (KPNC, KPSC) data, including birth dates, cancer diagnosis, and dates of death, to identify eligible patients. Eligible AYA patients were patients 12 to 39 years of age at death who were diagnosed with stage I through IV cancer and enrolled in KPSC or KPNC insurance plans or receiving care at DFCI more than 90 days prior to death. To focus on patients for whom end-of-life care planning may have been indicated, we included patients with stage IV cancer and those with stage I through III cancer and a new metastasis (*International Statistical Classification of Diseases, Ninth Revision* [*ICD-9*] codes for second malignant neoplasm of other organs, 197.0-197.8, 198.0-198.82, and 198.89; *ICD-10* codes, C78-C79), or likely recurrence, defined as more than 1 chemotherapy regimen with more than 90 days between episodes of administration.^20,21^ Recurrence or progression was confirmed via medical record review.

### Data Sources

Data were obtained from electronic health data and medical records from each site. At DFCI, an electronic clinical database, Oncology Data Retrieval System, was queried to identify eligible patients and sociodemographic and treatment information. Once patients were identified, medical records were used to obtain inpatient and outpatient documentation. KPNC and KPSC maintain Surveillance, Epidemiology and End Result–affiliated cancer registries with data on all patients diagnosed or treated for cancer at their sites during the study period. KPNC and KPSC also maintain medical records and clinical databases, including membership, diagnosis, procedures, pharmacy prescriptions or infusions, health care utilization, and outside claims.

Electronic clinical databases were used to ascertain patient sex; dates of birth, diagnosis, and death; cancer type and stage; and, when possible, race, ethnicity, and care and treatment received. Patient race and ethnicity were ascertained from the clinical database or electronic health record and were included in the analyses to fully characterize the cohort. Race categories followed the National Institutes of Health guideline and included the following categories: American Indian or Alaska Native, Asian, Black or African American, Native Hawaiian or Other Pacific Islander, White, more than 1 race, declined to state, not documented, and unknown. Ethnicity categories included Hispanic or Latino, not Hispanic or Latino, and not documented. Manual medical record review was conducted by abstractors at each site to abstract data on GOC conversations and documented treatment preferences. All reviewers were trained to use a detailed data abstraction algorithm. Initially 2 reviewers abstracted each medical record with weekly conference calls to resolve discrepancies until abstraction between reviewers showed higher than 95% agreement, then transitioned to monthly calls between abstractors to maintain consistency.^17^

### Measures

The GOC discussions and their dates were abstracted from available medical records. Medical record review focused on the last 90 days of life; however, some patients’ records in this time frame referenced discussions that happened earlier in care. In these cases, GOC discussions were documented as having occurred prior to the last 90 days of life. The GOC were categorized as a preference for (1) care focused on cure; (2) life-prolonging care, directed at life prolongation or using all possible measures; (3) care focused on quality of life, comfort, symptom management, or palliation; or (4) undecided preference, when goals were elicited but patients or family members did not specify a decision. To ensure consistency in categorization, documented text from medical records describing patient goals was abstracted for review by the study team.^22^ Each instance of a documented GOC discussion on a unique date was abstracted along with documented goals. We also used available records to ascertain care received using existing quality indicators for end-of-life cancer care, including chemotherapy in the last 14 days of life; more than 1 emergency department visit in the last month of life; inpatient hospitalization and intensive care unit stays in the last month of life; palliative care referrals; hospice use (any hospice use and hospice enrollment >7 days before death); and do-not-resuscitate orders.^4,8,23,24,25^

### Statistical Analysis

For analysis, the timing of documented GOC was classified as having taken place in the initial (>60 days before death), middle (31-60 days before death), or late (≤30 days before death) end-of-life period. During each period, patients were categorized according to documented discussions and preferences using the following categories: preference for palliation; preference for alternative, nonpalliative goals (including cure and life-prolonging care); a discussion took place but goals were not documented; or no discussion took place. If no new discussions took place within the period, then the prior categorization was carried over into the next period. For example, if a patient had documented palliative goals in the initial period, but no further discussions in the middle period, then goals were categorized as palliative in the middle period. If a new discussion or more than 1 discussion took place, then the preference documented latest (ie, closest to death) was considered the preference for that period.

A Sankey diagram was used to depict change in documented discussions and GOC during the last 90 days of life. Patients were classified into 5 categories based on their trajectories: (1) no discussion, for patients without any documented GOC discussion; (2) earlier palliative goals, with first documented palliative goals more than 60 days before death; (3) middle palliative goals, with first documented palliative goals 31 to 60 days before death; (4) late palliative goals, with first documented palliative goals 30 days or less before death; and (5) nonpalliative goals, for patients with documented GOC that reflected something other than palliation. We examined associations between trajectories and age category (12-24 years of age at death, to match the World Health Organization definition of AYA, vs 25-39 years if age), sex, race, ethnicity, and cancer type (hematologic cancer, solid tumor, or brain tumor) using Fisher exact tests, and separately using multinomial logistic regression, adjusted for all other factors as well as the year of death and site of care. We also used Fisher exact tests to assess differences in end-of-life care received according to documented discussions, goals, and their timing. A 2-sided *P* < .05 was considered statistically significant. All analyses were conducted using R statistical software, version 4.3.2 (R Project for Statistical Computing).

## Results

Among 1929 AYA patients included in the analyses, the mean (SD) age at cancer diagnosis was 28 (8) years, just over half were female (1049 [54.5%]), and 875 (45.5%) were male. Medical records identified most AYA patients as White (1184 [61.4%]); 5 (0.3%) were American Indian or Alaska Native, 227 (11.8%) were Asian, 157 (8.1%) were Black or African American, 14 (0.7%) were Native Hawaiian or Other Pacific Islander, 11 (0.6%) were more than 1 race, 293 (15.2%) did not have medical record documentation of race, and 38 (2.0%) were other race. Just over one-quarter of patients (514 [26.6%]) had ethnicity documented as Hispanic, 762 patients (39.5%) as not Hispanic or Latino, and 653 patients (33.9%) had medical records that lacked documented ethnicity (Table 1).

**Table 1.** Cohort Characteristics

Characteristic	Patients, No. (%) (N = 1929)
Age at diagnosis, y^a^	
Mean (SD)	28 (8)
Median (IQR)	30 (23-35)
Sex^a^	
Female	1049 (54.5)
Male	875 (45.5)
Race	
American Indian or Alaska Native	5 (0.3)
Asian	227 (11.8)
Black or African American	157 (8.1)
Native Hawaiian or Other Pacific Islander	14 (0.7)
White	1184 (61.4)
More than 1 race	11 (0.6)
Not documented	293 (15.2)
Other^b^	38 (2.0)
Ethnicity	
Hispanic or Latino	514 (26.6)
Not Hispanic or Latino	762 (39.5)
Not documented	653 (33.9)
Primary cancer site^a^	
Bone or soft tissue	264 (13.7)
Brain	170 (8.8)
Breast	265 (13.8)
Gastrointestinal tract	369 (19.2)
Genitourinary tract	214 (11.1)
Head, neck, or thyroid	72 (3.7)
Leukemia	164 (8.5)
Lung	85 (4.4)
Lymphoma	119 (6.2)
Melanoma or skin	67 (3.5)
Other	136 (7.1)
Stage at diagnosis^a^	
I-III	1119 (67.6)
IV	537 (32.4)
Site of care	
DFCI	703 (36.4)
KPNC	526 (27.3)
KPSC	700 (36.3)

Abbreviations: DFCI, Dana-Farber Cancer Institute; KPNC, Kaiser Permanente Northern California; KPSC, Kaiser Permanente Southern California.

^a^ Missing data: age at cancer diagnosis, n = 37; sex, n = 5; primary cancer site, n = 4; stage, n = 273.

^b^ Other races included declined to state and unknown.

The Figure shows the evolution in documented discussions and GOC during the last 90 days of life (eTable in Supplement 1 gives additional details). During the initial end-of-life period (>60 days before death), most patients (1364 [70.7%]) had no current or prior documentation of a GOC discussion, 139 patients (7.2%) had a documented preference for care focused on palliation, and 426 (22.1%) had documented care goals other than palliation, including cure, life prolongation, or undecided goals. During the middle end-of-life period (31-60 days before death), 969 AYA patients (50.2%) did not have a current or previous documented GOC discussion, 331 AYA patients (17.2%) had palliative goals documented in medical records, and 629 AYA patients (32.6%) had other nonpalliative documented GOC. During the late end-of-life period (≤30 days before death), 322 AYA patients (16.7%) did not have a documented GOC discussion before death, 1113 AYA patients (57.7%) had a documented preference for palliation, and 494 AYA patients (25.6%) had other nonpalliative documented goals.

**Figure.**
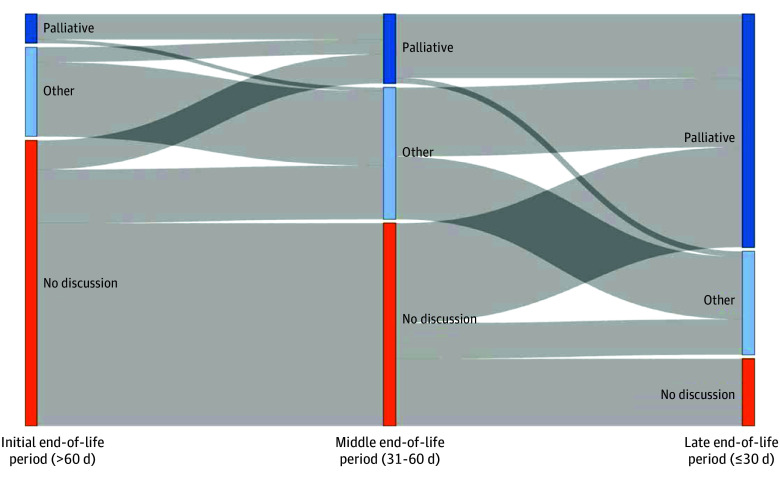
Trajectories of Change in Documented Discussions and Goals of Care During 3 End-of-Life Periods Palliative indicates palliative goals; other, nonpalliative goals (ie, curative, life-prolonging, or undecided goal); and no discussion, no documented discussion.

During the last 90 days of life, relatively few patients transitioned from palliative to nonpalliative GOC; 18 patients (0.9%) did so between the initial and middle end-of-life periods, and 26 patients (1.3%) did so between the middle and late end-of-life periods. More commonly, patients transitioned from nonpalliative goals to palliative goals (71 [3.6%]) from the initial to middle period; 322 [16.7%] from middle to late period) or from no documented discussion to palliative goals (129 [6.7%] from the initial to middle period and 478 [24.7%] from the middle to late period). Overall, 139 patients (7.2%) had an initial preference for palliation; 210 patients (10.9%) had a first preference for palliation expressed in the middle end-of-life period; and 800 patients (41.5%) first expressed palliative goals late, in the last month of life.

The trajectory of the change in goals varied by race, ethnicity, and cancer type (Table 2). Black and Hispanic or Latino patients were disproportionately represented among patients who had no documented GOC discussions compared with White and non-Hispanic patients (Black, 30 of 157 [19.1%] compared with White, 167 of 1184 [14.1%]; *P* < .001; Hispanic or Latino, 116 of 514 [22.6%] compared with non-Hispanic or Latino, 93 of 762 [12.2%]; *P* < .001). Asian patients, in contrast, were more heavily represented (28 [20.1%]) among patients who expressed palliative goals in the initial end-of-life period. Compared with patients with solid tumors or patients receiving neuro-oncology treatment, patients with hematologic cancers were disproportionately represented among patients who had no discussions (48 of 292 [16.4%] vs 244 of 1416 [17.2%] for solid tumor and 17 of 175 [9.7%] for brain tumor), late palliative goals (113 of 292 [38.7%] vs 594 of 1416 [41.9%] for solid tumor and 75 of 175 [42.8%] for brain tumor), and nonpalliative goals (97 of 292 [33.2%] vs 315 of 1416 [22.2%] for solid tumor and 40 of 175 [22.8%] for brain tumor) (Table 2).

**Table 2.** Characteristics of Patients According to Goals of Care and Their Timing

Characteristic	Patients, No. (row %)					*P* value^a^
	No discussion (n = 322)	Palliative goals			Nonpalliative goals (n = 458)	
		Initial (n = 139)	Middle (n = 210)	Late (n = 800)		
Age at death, y						
12-24	77 (16.8)	42 (9.2)	54 (11.8)	168 (36.7)	117 (25.5)	.10
25-39	245 (16.7)	97 (6.6)	156 (10.6)	632 (43.0)	341 (23.2)	
Sex						
Female	180 (17.2)	75 (7.1)	115 (11.0)	438 (41.8)	241 (23.0)	.90
Male	139 (15.9)	63 (7.2)	95 (10.9)	362 (41.4)	216 (24.7)	
Unknown	3 (60.0)	1 (20.0)	0	0	1 (20.0)	
Race						
Asian	45 (19.8)	28 (12.3)	32 (14.1)	88 (38.8)	34 (15.0)	<.001
Black or African American	30 (19.1)	8 (5.1)	17 (10.8)	68 (43.3)	34 (21.7)	
White	167 (14.1)	75 (6.3)	122 (10.3)	511 (43.2)	309 (26.1)	
Other race or not documented^b^	80 (22.2)	28 (7.8)	39 10.8)	133 (36.8)	81 (22.4)	
Ethnicity						
Hispanic or Latino	116 (22.6)	33 (6.4)	53 10.3)	217 (42.2)	95 (18.5)	<.001
Not Hispanic or Latino	93 (12.2)	57 (7.5)	96 (12.6)	324 (42.5)	192 (25.2)	
Not documented	113 (17.3)	49 (7.5)	61 (9.3)	259 (39.7)	171 (26.2)	
Cancer type						
Brain tumor	17 (9.7)	19 (10.9)	24 13.7)	75 (42.9)	40 (22.9)	<.001
Hematologic cancer	48 (16.4)	15 (5.1)	19 (6.5)	113 (38.7)	97 (33.2)	
Solid tumor	244 (17.2)	100 (7.1)	163 (11.5)	594 (41.9)	315 (22.2)	
Not specified	13 (28.3)	5 (10.9)	4 (8.7)	18 (39.1)	6 (13.0)	

^a^ *P* values are from Fisher exact tests.

^b^ Other races included: American Indian or Alaska Native, Native Hawaiian or Other Pacific Islander, more than 1 race, decline to state, and unknown.

Findings were similar in a multinomial regression model (Table 3) adjusted for age at death, year of death, sex, race, ethnicity, cancer type, and site of care. In addition to differences by race, ethnicity, and cancer type, older AYA patients (25 to 39 years of age) had lower odds of earlier palliative goals (odds ratio [OR], 0.79 [95% CI, 0.69-0.90]) and higher odds of late palliative goals (OR, 1.33 [95% CI, 1.09-1.61]) and nonpalliative goals (OR, 1.34, [95% CI, 1.09-1.65]) compared with younger AYA patients. The odds of all documented GOC also rose each year during the study period relative to no documented discussion (Table 3).

**Table 3.** Factors Associated With Goals and Timing^a^

Characteristic	Palliative goals						Nonpalliative goals	
	Initial		Middle		Late			
	OR (95% CI)	*P* value	OR (95% CI)	*P* value	OR (95% CI)	*P* value	OR (95% CI)	*P* value
Age at death, y								
12-24	1 [Reference]	NA	1 [Reference]	NA	1 [Reference]	NA	1 [Reference]	NA
25-39	0.79 (0.69-0.90)	<.001	1.08 (0.91-1.30)	.40	1.33 (1.09-1.61)	<.01	1.34 (1.09-1.65)	<.01
Year of death	1.07 (1.07-1.07)	<.001	1.11 (1.11-1.11)	<.001	1.14 (1.13-1.14)	<.001	1.13 (1.13-1.13)	<.001
Sex								
Female	1 [Reference]	.50	1 [Reference]	.40	1 [Reference]	.20	1 [Reference]	.06
Male	0.96 (0.83-1.10)		1.08 (0.90-1.29)		1.12 (0.95-1.33)		1.20 (0.99-1.46)	
Race								
Asian	1.75 (1.61-1.91)	<.001	1.38 (1.26-1.51)	<.001	0.82 (0.67-1.00)	.05	0.77 (0.69-0.85)	<.001
Black or African American	0.61 (0.59-0.62)	<.001	0.75 (0.72-0.78)	<.001	0.69 (0.58-0.83)	<.001	0.76 (0.68-0.85)	<.001
White	1 [Reference]	NA	1 [Reference]	NA	1 [Reference]	NA	1 [Reference]	NA
Other race or not documented^b^	1.39 (1.19-1.63)	<.001	1.23 (1.05-1.44)	.01	0.81 (0.65-1.02)	.07	1.26 (1.05-1.52)	.01
Ethnicity								
Hispanic or Latino	0.48 (0.42-0.55)	<.001	0.53 (0.45-0.61)	<.001	0.67 (0.56-0.81)	<.001	0.56 (0.47-0.66)	<.001
Not Hispanic or Latino	1 [Reference]	NA	1 [Reference]	NA	1 [Reference]	NA	1 [Reference]	NA
Not documented	0.90 (0.81-1.00)	.04	0.71 (0.64-0.78)	<.001	0.89 (0.75-1.06)	.20	1.18 (1.02-1.36)	.02
Cancer type								
Brain	1 [Reference]	NA	1 [Reference]	NA	1 [Reference]	NA	1 [Reference]	NA
Hematologic cancer	0.29 (0.27-0.31)	<.001	0.25 (0.23-0.28)	<.001	0.52 (0.47-0.58)	<.001	0.88 (0.78-1.00)	.06
Solid tumor	0.49 (0.44-0.56)	<.001	0.58 (0.50-0.67)	<.001	0.66 (0.58-0.77)	<.001	0.84 (0.73-0.97)	.02

Abbreviations: NA, not applicable; OR, odds ratio.

^a^ Multinomial logistic regression model adjusted for site, relative to adolescent and young adult patients with no documented discussion about goals of care.

^b^ Other races included: American Indian or Alaska Native, Asian Indian, Native Hawaiian or Other Pacific Islander, more than 1 race, decline to state, or unknown.

Discussions, care goals, and their timing were associated with end-of-life care received (Table 4). For example, chemotherapy in the last 14 days of life was received by 42 patients (13.0%) with no documented GOC discussion and 90 patients (19.7%) with nonpalliative GOC, relative to patients with palliative goals in the initial (9 [6.5%]), middle (11 [5.2%]), and late (107 [13.4%]) periods (*P* < .001). Similarly, care in the intensive care unit in the last month of life ranged from 12 patients (12.9%) among AYA patients with palliative goals in the initial end-of-life period to 272 patients (34.0%) with late palliative goals and 168 patients (36.7%) with nonpalliative goals (*P* < .001). Care patterns were similar for late life emergency department visits (17 patients [12.2%] with initial palliative goals vs 237 patients [29.6%] with late palliative goals and 110 patients [24.0%] with nonpalliative goals; *P* < .001) and hospitalizations in the last month of life (60 patients [43.2%] with initial palliative goals vs 629 patients [78.6%] with late palliative goals and 333 patients [72.7%] with nonpalliative goals; *P* < .001), with increasing utilization of each among patients with documented palliative GOC closer to death. Palliative care referrals, hospice referrals, and do-not-resuscitate orders were higher among AYA patients with palliative goals, with little variation based on the timing of goals, and lower for AYA patients with nonpalliative goals or no discussions (Table 4).

**Table 4.** Care Received According to Goals of Care Discussions, Documented Goals of Care, and Their Timing

Characteristic	Patients, No. (%)					*P* value^a^
	No discussion (n = 322)	Palliative goals			Nonpalliative goals (n = 458)	
		Initial (n = 139)	Middle (n = 210)	Late (n = 800)		
Chemotherapy in the last 14 d of life	42 (13.0)	9 (6.5)	11 (5.2)	107 (13.4)	90 (19.7)	<.001
ICU care in the last 30 d of life	89 (27.6)	18 (12.9)	42 (20.0)	272 (34.0)	168 (36.7)	<.001
>1 ED visit in the last 30 d of life	72 (22.4)	17 (12.2)	40 (19.0)	237 (29.6)	110 (24.0)	<.001
Hospitalization in the last 30 d of life	156 (48.4)	60 (43.2)	103 (49.0)	629 (78.6)	333 (72.7)	<.001
Palliative care referral	162 (50.3)	117 (84.2)	176 (83.8)	661 (82.6)	299 (65.3)	<.001
Hospice referral	156 (48.4)	114 (82.0)	174 (82.9)	561 (70.1)	199 (43.4)	<.001
Among patients in hospice, received hospice >7 d	113 (35.1)	94 (67.6)	154 (73.3)	350 (43.8)	146 (31.9)	<.001
Do-not-resuscitate order	111 (34.5)	121 (87.1)	178 (84.8)	692 (86.5)	260 (56.8)	<.001

Abbreviations: ED, emergency department; ICU, intensive care unit.

^a^ From Fisher exact tests.

## Discussion

In this cross-sectional study of AYA patients with cancer near the end of life, we found that records were increasingly likely to show palliative GOC over time, with 7.2% of the cohort noting palliative goals more than 60 days before death, 17.2% in the 31 to 60 days before death, and 57.7% in the last month of life. About one-fifth of the cohort transitioned from documented nonpalliative goals to a desire for palliative-focused care, perhaps reflecting changing awareness or acceptance of a poor prognosis.

Conversations about GOC are considered one of the fundamental interventions that clinicians can make to ensure that care reflects the values and preferences of patients living with advanced disease. However, these discussions happen inconsistently and often do not take place until late in life, in part because clinicians may hesitate to raise these topics earlier in care.^26,27,28,29^ This pattern creates a risk that care inadequately reflects patient preferences, with late discussions among older adults (aged 20 to >80 years) more often associated with use of intensive measures near the end of life.^30^

However, most previous work has measured patient preferences at a single point in time near death, rather than treating goals as a dynamic manifestation of patients’ illness understanding, values, and experiences. We sought to understand the nature of change in documented GOC for AYA patients near death as a possible window into goal evolution.

Most of the patients who expressed palliative goals late in life had never had a prior documented GOC discussion. Whether actual goals changed over this time period cannot be ascertained based on available records, an important limitation of using medical record review to understand this complex evolution. However, the increase in documented palliative GOC over time may also reflect clinician behavior, including a tendency to initiate discussions with AYA patients and family members late in life. GOC discussions are often delayed due to clinician concerns regarding increasing patient anxiety or contributing to loss of hope.^27,28^ Despite clinicians’ well-meaning concerns, evidence suggests that AYA patients value timely discussions regarding end-of-life care, with preferences for earlier discussions to best inform their decision-making and consideration of goals.^31,32,33^

Notably Black and Hispanic patients were more heavily represented among patients with no discussions compared with White and non-Hispanic patients, as were AYA patients with hematologic cancers compared with those with solid tumors or brain tumors. Extensive previous literature has identified disparities in end-of-life care for patients from historically minoritized racial and ethnic groups^34,35,36,37^ and for patients with hematologic cancers^38,39,40,41^; a lack of end-of-life discussions among these subpopulations creates a significant barrier to individualized, goal-concordant care. Consistent with existing quality measures, all patients should have the opportunity to discuss care preferences with their clinicians to ensure care reflects their personal values. These disparities present opportunity for intervention, which should include work to ensure that GOC conversations occur more regularly to meet the needs of a diverse population.

Discussions, goals, and their timing were also associated with end-of-life care received across all indicators examined, consistent with literature among older adults.^10,30^ Patients who had earlier documented palliative goals were less likely to receive intensive measures and more likely to receive palliative care and hospice than patients with nonpalliative goals, late palliative goals, or no discussion documented. For patients with nonpalliative goals, the use of intensive measures may reflect personal wishes for care and therefore be goal-concordant. However, patients with palliative goals expressed late, either in transition from nonpalliative goals or as the result of a first discussion, may miss opportunities to receive care consistent with their values focused on reducing suffering and enhancing quality of life.

From this study of AYA patients who died of cancer from 2003 to 2019, clinicians can draw 4 main conclusions. First, evolution in documented GOC was not uncommon. This may be because patients need opportunity to process information and reflect on their wishes or because care preferences depend on changing issues such as physical and psychosocial well-being or available options. Particularly for patients who express nonpalliative goals, ongoing discussions may support individualization of care and, for some, a transition toward palliative goals over time. Second, a small number of patients continued to express nonpalliative goals over time, and use of life-sustaining therapies may be goal concordant for this group. Third, we cannot know goals until we have discussions about them. Late GOC discussions risk goal-discordant care, especially for patients who desire care focused on palliation and quality of life. It is particularly important to recognize AYA patients at risk for disparate care at the end of life, including Black and Hispanic or Latino AYA patients and those with hematologic cancers, and for clinicians to create opportunities to discuss patient GOC. Fourth, repeated conversations about these topics take time. We commend clinicians who held ongoing discussions over time to make sure patient wishes were heard and understood. For AYA patients who are approaching the end of life, clinicians’ willingness to engage in hard conversations repeatedly can be an important gift.

### Limitations and Strengths

Limitations of this study include reliance on medical record documentation of GOC rather than on patient reports. Additional conversations may have taken place without documentation in medical records. In addition, documentation did not consistently describe who was present for the conversations or whether goals reflected the wishes of caregivers or the patients themselves. Finally, data were collected retrospectively from death, although clinicians must decide about the best timing for these complex end-of-life conversations prospectively in real time. Our findings were strengthened by inclusion of a diverse population from 3 sites with different care systems and supporting generalizability and by assessment of the longitudinal pattern of documented GOC over time.

## Conclusions

From this cross-sectional study of AYA patients who died of cancer from 2003 to 2019, palliative goals were rarely documented prior to the last month of life and were increasingly likely to be documented over time. Over the last 3 months of life, approximately 20% of the cohort transitioned from nonpalliative goals to palliative goals, although there was a small number of patients who continued to express nonpalliative goals throughout their care. As conversations about GOC occur inconsistently and often late in the end-of-life course, clinicians can improve clinical practice by offering to discuss the GOC of AYA patients with cancer earlier and by returning to these conversations over time to ensure the goals have not evolved. In particular, Black and Hispanic patients and patients with hematologic cancers were found to be at higher risk of lacking opportunities to hold these critical GOC discussions; thus, special attention should be paid to these populations to allow them to express their GOC and receive goal-concordant care.

## Notes

Supplement 1. **eTable.** Trajectories of change as depicted in the Figure; patterns show goals across the initial, middle, and late end-of-life periods based on when goals of care were first documented

Supplement 2.
Data Sharing Statement
