# Supplementary material for: Evolution in Documented Goals of Care at End of Life for Adolescents and Younger Adults With Cancer
Source: JAMA Netw Open. 2024 Dec 19;7(12):e2450489. doi: 10.1001/jamanetworkopen.2024.50489 (PMC11659917; doi:10.1001/jamanetworkopen.2024.50489)
Supplement: Supplement 1. — eTable. Trajectories of change as depicted in the Figure; patterns show goals across the initial, middle, and late end-of-life periods based on when goals of care were first documented [file jamanetwopen-e2450489-s001.pdf]

Supplementary Online Content

Mastropolo R, Cernik C, Uno H, et al. Evolution in documented goals of care at end of life for adolescents and younger adults with cancer. *JAMA Netw Open*. 2024;7(12):e2450489. doi:10.1001/jamanetworkopen.2024.50489

eTable 1. Trajectories of change as depicted in the Figure; patterns show goals across the initial, middle, and late end-of-life periods based on when goals of care were first documented

This supplementary material has been provided by the authors to give readers additional information about their work.

© 2024 Mastropolo R et al. *JAMA Network Open*.

eTable 1. Trajectories of change as depicted in the Figure; patterns show goals across the initial, middle, and late end-of-life periods based on when goals of care were first documented.

Group	Pattern[a]	Patient Number (N)	Percentage (%)
Initial palliative goals	P O O	10	0.5
	P O P	8	0.4
	P P O	7	0.4
	P P P	114	5.9
Middle palliative goals	N P O	10	0.5
	N P P	129	6.7
	O P O	9	0.5
	O P P	62	3.2
Late palliative goals	N N P	478	24.8
	N O P	130	6.7
	O O P	192	10.0
Non-palliative goals	N N O	169	8.8
	N O O	126	6.5
	O O O	163	8.4
No documented discussion about goals of care	N N N	322	16.7
		1929	100.0

P=palliative goals
O=non-palliative goals (curative, life-prolonging, or undecided goal)
N=no documented discussion

[a]The 1st, 2nd and 3rd letters in this column indicate the goals in 61-90 days, 31-60 days, and 0-30 days prior to the death, respectively.

© 2024 Mastropolo R et al. *JAMA Network Open*.
