# Supplementary material for: Evolution in Documented Goals of Care at End of Life for Adolescents and Younger Adults With Cancer
Source: JAMA Netw Open. 2024 Dec 19;7(12):e2450489. doi: 10.1001/jamanetworkopen.2024.50489 (PMC11659917; doi:10.1001/jamanetworkopen.2024.50489)
Supplement: Supplement 2. — Data Sharing Statement [file jamanetwopen-e2450489-s002.pdf]

Data Sharing Statement

Mastropolo. Evolution in Documented Goals of Care at End of Life for Adolescents and Younger Adults With Cancer. *JAMA Netw Open*. Published December 19, 2024. doi:10.1001/jamanetworkopen.2024.50489

Data
Data available: Yes
Data types: Deidentified participant data, Data dictionary
How to access data: Please contact the corresponding author for details of data sharing. Data will be shared in compliance with Dana Farber Cancer Institute, Kaiser Permanente, and NIH policies.
When available: With publication

Supporting Documents
Document types: None

Additional Information
Who can access the data: Researchers whose proposed use of the data has been approved
Types of analyses: Relevant analyses appropriate to the available data
Mechanisms of data availability: After approval of a proposal and with a signed data access agreement
